# Repression of the lysogenic P_R_ promoter in bacteriophage TP901-1 through binding of a CI-MOR complex to a composite O_M_-O_R_ operator

**DOI:** 10.1038/s41598-020-65493-0

**Published:** 2020-05-26

**Authors:** Margit Pedersen, Jesper Tvenge Neergaard, Johan Cassias, Kim Krighaar Rasmussen, Leila Lo Leggio, Kim Sneppen, Karin Hammer, Mogens Kilstrup

**Affiliations:** 1 University of Copenhagen, Department of Biology, Copenhagen, DK2200 Denmark; 2 Technical University of Denmark, Department of Biotechnology and Biomedicine, Lyngby, DK2800 Denmark; 3 University of Copenhagen, Department of Chemistry, Copenhagen, DK2200 Denmark; 4 University of Copenhagen, Center for Models of Life, Copenhagen, DK2200 Denmark

**Keywords:** DNA-binding proteins, Bacteriophages, Transcriptional regulatory elements

## Abstract

A functional genetic switch from the lactococcal bacteriophage TP901-1, deciding which of two divergently transcribing promoters becomes most active and allows this bi-stable decision to be inherited in future generations requires a DNA region of less than 1 kb. The fragment encodes two repressors, CI and MOR, transcribed from the P_R_ and P_L_ promoters respectively. CI can repress the transcription of the *mor* gene at three operator sites (O_R_, O_L_, and O_D_), leading to the immune state. Repression of the *cI* gene, leading to the lytic (anti-immune) state, requires interaction between CI and MOR by an unknown mechanism, but involving a CI:MOR complex. A consensus for putative MOR binding sites (O_M_ sites), and a common topology of three O_M_ sites adjacent to the O_R_ motif was here identified in diverse phage switches that encode CI and MOR homologs, in a search for DNA sequences similar to the TP901-1 switch. The O_R_ site and all putative O_M_ sites are important for establishment of the anti-immune repression of P_R_, and a putative DNA binding motif in MOR is needed for establishment of the anti-immune state. Direct evidence for binding between CI and MOR is here shown by pull-down experiments, chemical crosslinking, and size exclusion chromatography. The results are consistent with two possible models for establishment of the anti-immune repression of *cI* expression at the P_R_ promoter.

## Introduction

Genetic switches are used by temperate bacteriophages to decide between their two alternative life cycles following injection of their DNA into the host bacterium^1^; the lytic cycle where the phage multiplies and kills the bacterium at once or the lysogenic cycle where killing is suspended. In the lysogenic life cycle of most phages, the lytic functions are silenced by a CI repressor, which also renders the host cell immune to subsequent attacks by phages with homologous repressors (superinfection immunity).

**Figure 1:**
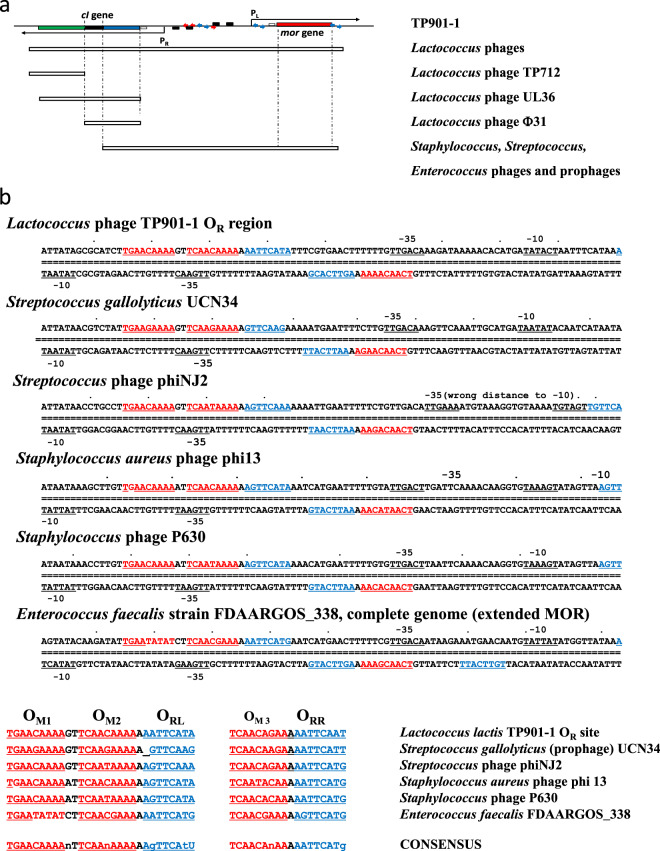
Regions with homology to the TP901-1 switch region at the nucleotide level. The nucleotide sequence of the switch region from bacteriophage TP901-1 was used in a search for similar sequences using the PSI BLAST web server. (**a**) Physical outline of the switch. Three different domains in the CI repressor are indicated by colors: N-terminal domain encoding DNA binding site (blue box, aa 1 to 80); middle CT1-domain encoding a flexible linker and a putative hook domain for tight dimerization (black box, linker aa 81 to 89, hook domain aa 90 to 121); C-terminal domain encoding multimerization sites (green box, aa 122 to 180). The *mor* gene is shown in red. Promoters P_L_ and P_R_ are shown as arrows and black boxes symbolizing −10 and −35 regions. Operator sites are shown as blue divergent arrows. White boxes in lower panel indicate areas of homology to the TP901-1 switch region found in genomes of various types of phage and pro-phage. (**b**) Top panel shows the nucleotide sequence of the O_R_ region. The two half sites of the O_R_ operator recognized by the N-terminal domain of the CI repressor are shown in blue. Putative O_M_ operators, numbered O_M1_, O_M2_, and O_M3_ from left to right, are shown in red. Promoter −10 and −35 motifs are underlined and marked, with the P_L_ (lytic) promoter transcribing towards right and the P_R_ promoter towards left. (**b**) bottom panel shows an alignment of all O_R_ half sites and O_M_ sites, with consensus sequences for each type.

Lactococcal bacteriophage TP901-1, belonging to the industrially important P335 family of phages, appears to have a simple decision switch mechanism^2–5^ involving two competing repressors CI and MOR (Modulator Of Repression). The genes for CI and MOR are divergently transcribed from two promoters (P_R_ and P_L_, respectively) located back to back (see Fig. 1) in almost perfect symmetry around a central operator (O_R_) recognized by CI^4,6^. Overlapping the lytic *mor* (P_L_) promoter is a second CI operator (O_L_), and a third (O_D_) is located in the distal part of the *mor* gene. This overall operator topology appears to be shared among many lactococcal and streptococcal bacteriophage switches, despite the fact that the operator sequences do not follow the same consensus^7^. The CI monomer consists of an N-terminal DNA binding region^8^, a helical hook region which mediates dimerization^9^ and a C-terminal helical region of unknown structure necessary for multimerization^3^. Upon binding of CI dimers to each of these operators in the lysogenic state of the TP901-1 switch, the P_L_ promoter is almost fully repressed ensuring low concentrations of MOR and lytic proteins, while the P_R_ promoter is only partially auto-repressed resulting in an optimal CI concentration^10^. In the lytic state of the switch, the P_R_ promoter is more severely repressed by MOR dependent repression while the P_L_ promoter is de-repressed. The apparent simplicity of the system is however deceiving as genetic analysis has revealed that the CI protein is required in collaboration with MOR for repression of its own promoter^3^, so the *cI* gene needs to be transcribed at a certain level in the lytic state of the switch. A helix-turn-helix (HTH) DNA binding domain has been identified in the *mor* open reading frame, but MOR is apparently unable to bind efficiently to DNA by itself^3^. A model for MOR dependent repression of the P_R_ promoter has hypothesized that a complex between CI and MOR is formed in solution^4,11^ and that this CI:MOR complex binds to an O_M_ operator site located downstream from the P_R_ promoter preventing transcription of the *cI* gene^3^. To explain the lack of repression of the P_L_ promoter in the lytic state, binding of MOR to CI must prevent binding to the O_R_, O_L_, and O_D_ operator sites. Thus according to the TP901-1 switch model, MOR functions as anti-repressor of CI while CI functions as co-repressor for MOR in the lytic state.

Anti-repressors against C repressors are fairly common in phage switches for use in the lytic induction of lysogenic pro-phages, such as the Ant protein in P22^12^ and the SOS-induced Tum anti-repressor in phage 186^13,14^. Anti-repressors have also been detected in decision switches, as exemplified by the Coi anti-repressor protein in bacteriophage P1^15^. An anti-repressor function of MOR in the TP901-1 decision switching is therefore not unprecedented. Co-repressor proteins have also been detected among decision switches as the Lxs CI-co-repressor in bacteriophage P1^16,17^. The suggestion that CI should function as a co-repressor for MOR is therefore not exceptional, and would add an interesting twist to the P1 paradigm.

In the present report we provide evidence for the last part of the TP901-1 switch model, i.e., that CI is co-repressor for MOR in the lytic state. Repression of the lysogenic P_R_ promoter is obtained by binding of a CI-MOR complex to a composite binding site including the O_R_ operator and flanking O_M_ binding sites. While linear multimeric repressor structures are known to be stabilized by binding to stretches of operators, as exemplified by the Cox repressor in P2^18,19^ and the Apl repressor in phage 186^20^, we believe that this is the first report of a repressor structure in which two competing repressors are collaborating in stabilization of a common repressing complex for the benefit of the one. We supply bioinformatic evidence that such composite operator structures are present in the switches of a large and diverse group of phages using CI/MOR homologues including phages of human pathogens.

Analysis of decision switching has been possible due to the use of our unique switch plasmids, such as pMAP50^3^ that enables the easy quantification of decision switching frequencies by plating transformants with the transcriptional fusion plasmids directly on agar plates containing X-gal. Switch plasmids also have the benefit in comparison with complete phage models that both the lysogenic (immune) state and the lytic (anti-immune) state are viable and may be subsequently analyzed^3^.

## Results and discussion

A homologue of the entire switch module from bacteriophage TP901-1 was previously detected in prophage bIL285 in the *Lactococcus lactis* subspecies *lactis* laboratory strain IL1403^21^. In the *Lactococcus lactis* laboratory strains MG1363^22^ belonging to subspecies *cremoris* we have likewise found a small region corresponding the C-terminal domain of the CI repressor in the T712 prophage of MG1363 (data not shown). Since homologues of the TP901-1 switch exist even in the two most widely used laboratory strains it became interesting to do a BLAST search^23^ in the entire NCBI nucleotide collection for phages and pro-phages using closely related switch regions. A selection of very informative sequences (see Fig. 1) resulted from such a search for DNA regions with extensive similarity to the functional switch region present in switch plasmid pMAP50. All sequences corresponded to genomes of phages or pro-phages with *Lactococcus, Streptococcus, Enterococcus*, or *Staphylococcus* hosts.

A number of *Lactococcus* phages and pro-phages contained entire switch regions with extensive homology throughout the switch region and must be expected to use identical mechanisms in their decision switching as TP901-1 (e.g., pro-phages in *L. lactis* strains A76 (subsp. *cremoris*), A12, UC77, IL1403, 229 (all subsp. *lactis*), and phage bIL285). Of more interest, however, was the identification of a large number of streptococcal, staphylococcal, and enterococcal phages and pro-phages that showed homology to the *mor* gene and the intergenic region, but only to the first part of the *cI* gene encoding the N-terminal domain, known to contain DNA binding domain. Beyond the N-terminal domain no similarity was detected to the TP901-1 *cI* gene for these phages (see Fig. 1a).

When the non-homologous C-terminals of these CI repressors were analyzed for known protein motifs, they were found to encode domains with high homology to more classic CI repressors^24^, which perform RecA dependent auto cleavage. Curiously, it has been shown that the CI repressor from TP901-1 does not seem to auto-cleave^25^, and no peptidase domain can be detected in the primary sequence.

### A composite O_M1_-O_M2_-O_R_-O_M3_ operator complex is preserved in homologous phage switches

**Figure 2:**
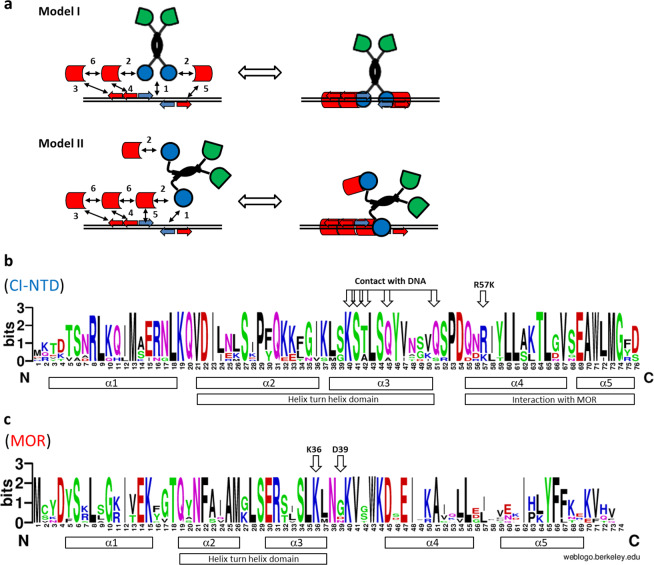
Models for binding of CI_2_-Mor complexes to a composite O_M1_-O_M2_-O_R_-O_M3_ operator, and functional domains in CI and MOR proteins. (**a**) In the absence of MOR, CI_2_ binds in its native conformation to the O_R_ site. Sufficient concentrations of CI and MOR result in MOR:CI_2_ binding that lowers the affinity of Cl_2_ towards O_L_, O_D_, and O_R_. Model I) MOR binds on the outside of CI_2_ which has the advantage that it enables a straight forward binding of a MOR-MOR-CI_2_-MOR complex to the composite O_M1_-O_M2_-O_R_-O_M3_ operator, but the drawback that prevention of CI_2_ binding to O_L_, O_D_, or O_R_ is dubious. Model II) MOR binds on the inside of the “legs” of the CI dimer, which has the advantage that it prevent CI_2_ binding to O_L_, O_D_, and O_R_. This model however has the drawback that it requires binding between a MOR-MOR-MOR-CI_2_ complex and a composite O_M1_-O_M2_-O_R_ operator. Binding of the protein complexes to the composite O_M1_-O_M2_-O_R_-O_M3_ operator is stabilized by a number of protein:DNA and protein:protein interactions, each supplying binding energies, as represented by numbered arrows in the left panels. b,c) LOGO plots was created using the http://weblogo.berkeley.edu/logo.cgi web based service, using an alignment of CI N-terminal Domain (**b**) or MOR (**c**) sequences from bacteriophages containing Switch regions with homology to the TP901-1 O_R_ region and O_M1_-O_M2_-O_R_-O_M3_ topology (shown in Fig. 1). (**b**) Above the LOGO plot sequence the residues involved in CI-DNA binding is shown with arrows, as well as the location of a conservative R57K substitution previously identified to give a clear plaque phenotype. (**c**) Above the LOGO plot sequence the invariant K36 (MOR numbering) and variant D39 are shown as arrows. Most of the DNA binding HTH domain is conserved, with an α3 recognition helix motif ERxxSLK (E30 to K36), a turn motif, xLS (G27 to S29), and a stabilizing α2 helix domain QxNFxxAM (Q19 to M26). Aside from this an N-terminal α1 helix motif SxLxGxIxEK (S6 to K15) and a conserved central α4 helix motif DxEIxxAxxxL (D45 to L65) is shown.

Since the DNA binding region of the CI repressor is located in the N-terminal domain of the phages and pro-phages identified above, the possibility remained that the homologous intergenic regions could reveal similarities relating to CI and MOR operator sites. Therefore, we did a manual curation of the intergenic regions from switches where homology spans the *mor* gene, the intergenic region, and the first part of the *cI* gene encoding the N-terminal domain. In this process a central CI operator sequence, corresponding to the O_R_ site from TP901-1 (see Fig. 1b), was identified in the intergenic regions from *Staphylococcus, Streptococcus* and *Enterococcus* switches. All regions contain putative P_L_ and P_R_ promoters, following the normal promoter geometry and fitting the −10 and −35 consensus sequences perfectly. Previously we had considered the A-rich sequences flanking the O_R_ sites as possible MOR binding sites, and the comparison in Fig. 2 shows that a [TCAACAnAA] or [TCAAnAAAA] motif is situated adjacent to the 5′-end of each O_R_ half site in all the CI-MOR switches, with a spacing of one A nucleotide. Furthermore an extra motif [TGAACAAAA] is present on the P_R_-proximal site, spaced by two nucleotides (GT or AT).

If the motifs (shown in red in Fig. 1b) serve as binding sites for MOR binding in the CI-MOR anti-immune complex, then the most straight forward binding mode (**Model I** in Fig. 2a) would be a MOR-MOR-CI_2_-MOR complex that binds linearly to the O_M1_-O_M2_-O_R_-O_M3_ operator complex and result in asymmetrical repression of the P_L_ and P_R_ promoters. Repression would be most severe for P_R_, as MOR-MOR binding to the O_M1_-O_M2_ operators might compete efficiently with RNA polymerase binding to this promoter. This model, however, has the twist that prevention of CI_2_ binding to the O_L_ and O_D_ operators will require a conformational change that prevents binding to O_L_ and O_D_ upon binding of MOR outside of the CI_2_ dimer, but which still allows CI_2_ binding to O_R_.

A second possibility which addresses this issue could be proposed in which the MOR protein binds to the CI_2_ dimer in a way that prevents normal CI_2_ binding to its operators (**Model II** in Fig. 2a). This geometry would have the benefit that it forces the two DNA-binding motifs on CI_2_ far apart, and prevent binding to O_L_, O_D_, or O_R_. However, the prevention of CI_2_ binding in Model II sacrifices the simplicity of binding to the composite operator. If Model II is right this will require that the CI-proximal MOR is stabilized by binding to the left O_R_ half-site (O_RL_, see also Fig. 2a), which should then function as a pseudo-O_M_ site, resulting in binding of a MOR-MOR-MOR-CI_2_ complex to the O_M1_-O_M2_-O_RL_-O_RR_ operator.

Model I and Model II have their pros and cons, and each rely on the importance of the O_M_ sites identified by the phylogenetic analysis, and that these actually serve as MOR binding sites. To distinguish between the two models we will need to acquire much more information about the detailed interaction of MOR and CI. However, in support of either model, we will supply experimental *in vivo* evidence for the importance of the O_R_, O_M1_, O_M2_, and O_M3_ sites for the anti-immune repression of the P_R_ promoter, as well as *in vitro* evidence of the CI-MOR, and MOR:MOR interactions that are functional prerequisites for all three models.

### Mutation of the O_R_ site decrease anti-immune repression of the P_R_ promoter

For quantification of the importance of the O_R_ operator in the anti-immune repression of P_R_, the anti-immune state had to be secured in the *L. lactis* laboratory strain MG1363^22^. Technically we needed to establish conditions where the P_L_ promoter had won the “decision race” and resulted in sufficient levels of both CI_2_ and MOR to form the putative CI:MOR complex for binding to the OM_1_-OM_2_-O_R_-OM_3_ operator and secure repression of the P_R_ promoter. Plasmid pAB223^2^ contains the full switch region from TP901-1 carried on the low-copy number plasmid pCI3340^26^ and is capable of establishing either the anti-immune phenotype described above, or the immune state where CI dimers in the absence of MOR bind to the O_L_, O_R_, and O_D_ operators and repress the P_L_ promoter^2^. Transformation of MG1363 derivatives with pAB223 has previously been shown to produce approximately 94% transformants in the anti-immune state with repressed P_R_ levels, and 6% in the immune state with unrepressed P_R_ levels^2^. Since strains carrying “switch plasmids” are phenotypically stable with respect to the switching state, we analyzed only a small number of MG1363/pAB223 transformants. Candidate MG1363/pAB223 transformants were subsequently transformed with the P_R_-*lacLM* fusion plasmid pJT2 (see Table 1). Plasmid pJT2 is based upon the integrative fusion plasmid pLB86^27^ and contains the intergenic region between the *cI* and *mor* genes from TP901-1 inserted upstream from the *lacLM* reporter genes. It includes both promoters and the O_R_ and O_L_ operators, but no functional phage genes. One positive transformant with severely repressed P_R_ levels was selected and named JT2. The expression of β-galactosidase from this plasmid was approximately 40-fold elevated in the absence of pAB223 (see below), showing that the JT2 switch phenotype was indeed repressing for the P_R_ promoter.

**Table 1:** Importance of the O_R_ operator for anti-immune repression of the P_R_ promoter in the TP901-1 switch.

Fusion plasmid {genetic elements present}	Mutation	β-galactosidase activity (differential synthesis rates)		
		P_R_ level in JT2 (SD)	P_R_ level in LB504 (SD)	Repression fold
pJT2 *lacLM* {←P_R_ O_M1_ O_M2_ O_RL_ O_RR_ O_M3_ P_L_ →O_L_}	None	0.17 (0.086)	6.6 (4.2)	40
pJT3 *lacLM*{←P_R_ O_M1_ O_M2_ O_RL_ [mut] O_M3_ P_L_→O_L_}	O_R_: From AGTTCACG To AGGATCCG	0.45 (0.53)	1.8 (1.7)	4
pJT5 *lacLM*{←P_R_ O_M1_ O_M2_ O_R_ O_M3_}	None	0.20 (0.069)	4.3 (3.4)	20
pJT13 *lacLM*{←P_R_ [mut] O_M2_ O_R_ O_M3_}	O_M1_: From TGAACAAAA-GT To TGAAC**TCC**A-GT	3.6 (0.31)	5.9 (0.097)	2
pMK1216 *lacLM*{←P_R_ O_M1_ [mut] O_R_ O_M3_}	O_M2_: From TCAACAAAA-A To TCAAC**CGTG**-A	0.37 (0.072)	2.8 (0.52)	8
pMK1217 *lacLM*{←P_R_ O_M1_ O_M2_ O_R_ [mut]}	O_M3_: From TCAACAAAA-A To TCAACAA**GT**-**T**	0.82 (0.23)	2.1 (0.48)	3
pJT6 *lacLM*{←P_R_ O_M1_ O_M2_ O_R_}	O_M3_ deletion	0.46 (0.24)	1.3 (0.91)	3

Expression levels from P_R_-*lacLM* reporter fusions in switch background (JT2) compared to a genetic background without CI and MOR (LB504).

As mentioned above the binding of CI_2_ to the O_R_ operator is important for the anti-immune repression of P_R_ in either model. Therefore we constructed a mutant plasmid pJT3 where the right O_R_ half site (O_RR_) was changed from [AGTTCACG] to [AGGATCCG] and creating a BamHI restriction site, identical to a published O_R_ mutation^3^. pJT2 and pJT3 were integrated as single copy fusions into the chromosomal *attB* site in JT2 (MG1363/pAB223 [anti-immune]) as well as in LB504 (MG1363/pLB65 [no CI or MOR present]). The average β-galactosidase levels, measured in biological triplicates, are shown in Table 1. It is clear that the mutation of the O_R_ site severely reduces the anti-immune repression of the P_R_ promoter, suggesting that the O_RR_ site is important for the anti-immune repression. This conclusion appears to be in direct contradiction with a previous report^3^, where the exact same mutation was shown not to prevent anti-immune P_R_ repression when it was present in the switch plasmid. Without going into a detailed discussion, the apparent contradiction may be explained by a 7-fold elevated MOR expression in the O_R_ mutant under anti-immune conditions that could increase the formation of the CI:MOR complex and the extent of P_R_ repression.

### Mutation of the O_M1_, O_M2_, and O_M3_ sites decrease anti-immune repression of the P_R_ promoter

After pointing at the importance of the O_RR_ half site for the anti-immune repression of P_R_, the involvement of the putative O_M1_, O_M2_, and O_M3_ sites was tested using a library of random PCR generated mutations for each O_M_ site targeting the A-rich areas. A selection of such mutants were measured as single copy fusions integrated into the chromosomes of JT2 and LB504, three of which (pJT13, pMK1216, and pMK1217) are shown in Table 1. To avoid interference with the O_L_ operator and the P_L_ promoter, a fusion plasmid pJT5 devoid of these elements was used for comparison between mutated and wildtype O_M_-sites. Plasmid pJT5 showed 20-fold repression and similar expression levels as pJT2 under both anti-immune and unrepressed conditions, showing that the interference between the two promoters can be neglected.

It is clear from the results in Table 1 that mutations in each of the three putative O_M_ operators diminish the anti-immune repression of the P_R_ promoter. It appears as if the O_M2_ site (with four consecutive mutated bases) is less crucial for full repression compared to O_M1_ and O_M3_ (with three and two mutated bases, respectively), which could reflect the increased stabilization of a MOR protein that is sandwiched between two MOR proteins (Model II) or between MOR and CI (Model I). Deletion of the entire O_M3_ operator (pJT6 in Table 1) has a similar effect as mutating the A-rich region (compared with pMK1217). Collectively, these data confirm our phylogenetic evidence about the importance of the putative O_M_ sites for establisment of anti-immune repression, presumably as operators for the CI:MOR anti-immune complex formation.

Due to an unusual and interesting response of the P_R_ promoter to the optical density of the culture (see supplementary material) we had to restrict our sampling to cell densities with OD_450_ values in the range between 0.25 and 0.75 to get the best determinations. When quantifying P_R_ repression in Table 1 the β-galactosidase activity was measured as the differential synthesis rate from three sample points within the restricted OD values and calculated as Δ(total enzyme activity)/Δ(OD450). It is highly interesting that the P_R_ promoter shows this cell density or growth phase dependence, which resembles a quorum sensing response except for the low magnitude of the induction, but so far our analysis has not shed light on the mechanisms behind the dependence.

### A DNA binding domain in the MOR protein is important for establishment of the anti-immune state

Above we showed that disrupting O_RR_, O_M1_, O_M2_, or O_M3_ had severe implications for establishment of a strongly repressing anti-immune complex, which we take as evidence for the importance of CI binding to the O_RR_ half site and MOR binding to the three O_M_ operators. In the following, we analyze the importance of a putative DNA binding site in the MOR protein for the establishment of the anti-immune state.

Previously a HTH domain between residues Q19 and N38, with close resemblance to DNA binding domains (see α2 and α3 in Fig. 2c) has been reported in the primary sequence of the MOR protein^2^. To analyze whether this putative DNA binding domain is important for the establishment of the anti-immune state, a conserved K36 residue of the recognition helix was changed to an alanine along with a D39A substitution. To analyze the importance of the K36 and D39 residues in the MOR protein, we compared the switching frequencies of two switch plasmids, one with a wild type *mor* gene (pAB223) and one with a mutated *mor* gene producing the MOR K36A D39A variant (pMAP109). Because the two switch plasmids did not contain reporter genes, the switch status was monitored with a P_L_-*lacLM* promoter fusion present in the recipient strain (AJ189). When plasmid pMAP109 was used to transform AJ189, and transformants were selected on solid media containing erythromycin and X-gal, only white transformants were obtained (data not shown), while transformation with pAB223 resulted in the expected 90 to 95% blue (anti-immune) colonies, showing that the putative DNA binding domain in MOR is important for establishment of the anti-immune complex.

### Evidence for CI:MOR interaction by pull down experiments

**Figure 3:**
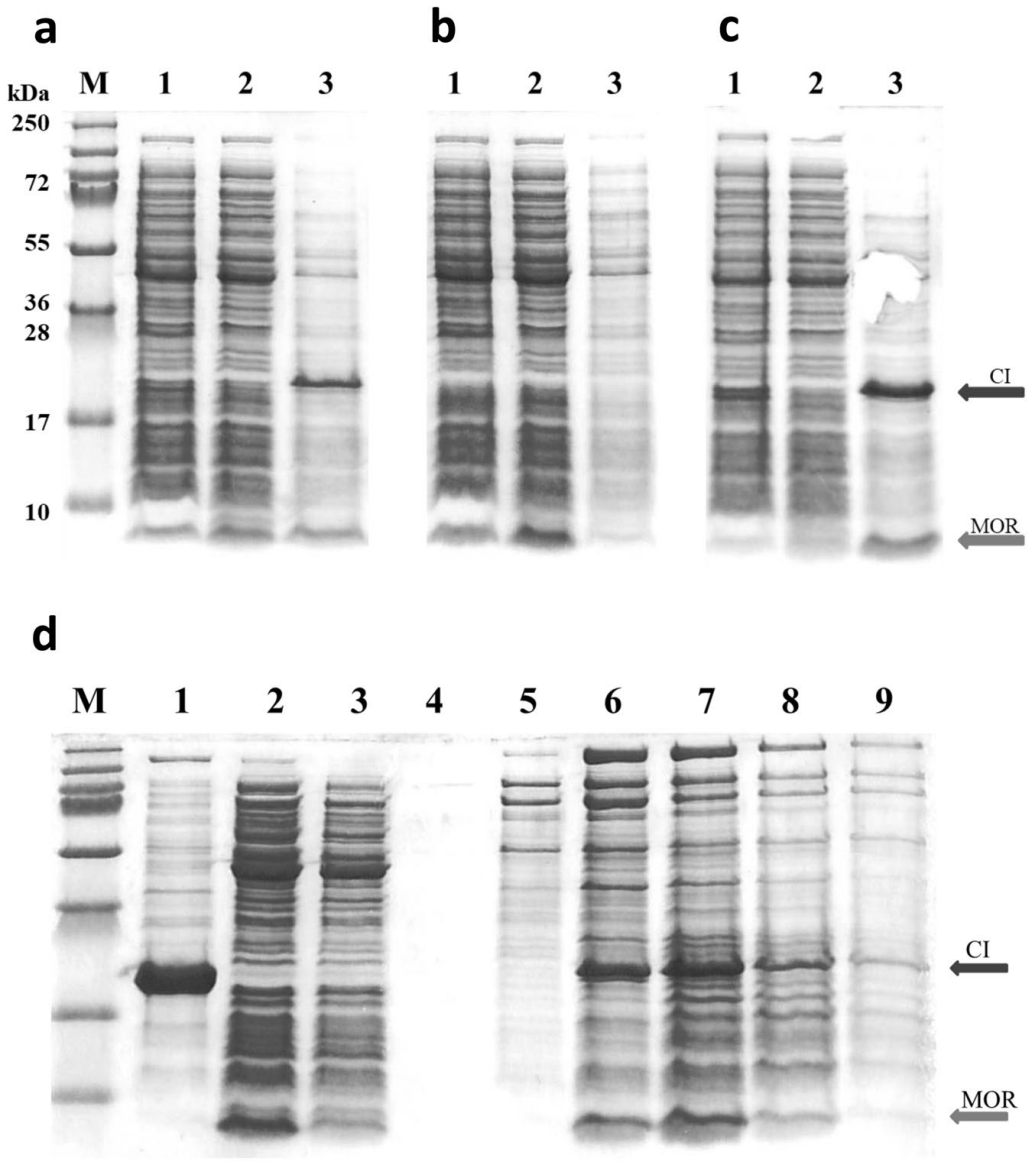
Pull-down experiment using unspecific CI binding to chromosomal DNA or heparin. *E. coli* cells expressing CI (AJ159, **a** and **c**) or MOR (AJ172, **b** and **c**) were disrupted by sonication, either separately (**a**,**b**) or together (**c**), and centrifuged. Samples were withdrawn after sonication (lane 1), from the supernatant after centrifugation (lane 2), and from the pellet after elution of CI with 1 M NaCl (lane 3). Proteins were solubilized in SDS buffer and separated by SDS PAGE, followed by coomassie staining. Lanes marked M contained protein weight standards with molecular weights as shown left of each band. (**d**) CI and MOR extracts were prepared from overexpressing *E. coli* cells (AJ159 and AJ172, respectively) after sonication and centrifugation. MOR protein extracts were sampled from the supernatant after centrifugation, while CI extracts were obtained by elution of CI from the centrifugation pellet in 1 M NaCl. Lane 1) Binding of CI extract to a heparin column (CI:heparin) followed by elution with 1 M NaCl. Lane 2) Run-through from binding of a MOR extract on a heparin column. Lane 3) Run-through from binding of a MOR extract to a CI:heparin column. Lanes 4 to 9) Elution of bound CI and MOR from a CI:heparin column with 1 M NaCl, in small fractions. Proteins were solubilized in SDS buffer and separated by SDS PAGE, followed by coomassie staining. Lanes marked M contained protein weight standards.

Interactions between CI and MOR, which are crucial for both model I and II was analyzed by a simple pull-down experiment where we took advantage of the strong salt sensitive unspecific DNA binding of CI, previously used in the purification of the native CI repressor^28^. When CI is expressed from an expression plasmid in *E. coli* strain AJ159, the entire pool of CI protein becomes bound to the chromosomal DNA after cell disruption (see Fig. 3a, lane 1), and is subsequently pulled down during centrifugation (Fig. 3a, lane 2). Suspension of the pellet containing DNA and CI in a 1 M NaCl solution results in elution of almost pure CI (Fig. 3a, lane 3). The DNA binding ability of CI was well known, but it was interesting whether the DNA binding motif on MOR, that was found to be important for establishment of the anti-immune CI:MOR repression (see above), could bind DNA so strongly that the MOR protein would precipitate with *E. coli* DNA. Expression of MOR protein on its own (i.e. in the absence of CI) from an expression plasmid in *E. coli* AJ172 (Fig. 3b, lane 1) was shown to result in a high MOR concentration only in the supernatant (Fig. 3b, lane 2), yet not in the DNA pellet following centrifugation (Fig. 3b, lane 3). In contrast, pull-down of MOR by binding to DNA-bound CI was in contrast very efficient. When an extract of AJ159 cells containing high concentrations of expressed CI protein was mixed with an extract of AJ172 cells containing high concentrations of expressed MOR protein (Fig. 3c, lane 1), both CI and MOR ended up in the pellet after cell disruption and centrifugation (Fig. 3c, lanes 2 and 3). This shows that MOR is pulled down from solution by the DNA-bound CI protein.

Interestingly, pull-down experiments using extracts of CI-overexpressing AJ159 cells mixed with extracts from MP1047 cells overexpressing a MOR[K36A D39A] recombinant protein also result in efficient pelleting of both proteins (data not shown). This shows that the CI-MOR interaction does not require that MOR has a native DNA binding domain, and must dependent upon strong CI-MOR binding that does not interfere with CI unspecific DNA binding.

A similar result was found when a heparin column was used to monitor binding of CI and MOR from cellular extracts (see Fig. 3d). Heparin is a polymer with a negative charge distribution that mimics the DNA backbone and is used in affinity chromatography to select for DNA binding proteins. CI was bound efficiently on the heparin column (Fig. 3d, lane 1). In contrast all MOR protein could only be found in the run-through when it was loaded on a pure heparin column (Fig. 3d, lane 2). However, if the column had been allowed to interact with purified CI, forming a complex between heparin and the CI DNA binding domain before addition of MOR extract, no MOR could be found in the run-through (Fig. 3d, lane 3). When CI binding to heparin was disrupted with 1 M NaCl, both CI and MOR was found in fraction 3 to 5 (Fig. 3d, lanes 6 to 8). This again confirms that the intrinsic DNA binding of MOR is too weak for affinity purification, but that CI:MOR interactions are strong enough to permit retention of a CI:MOR complex when CI is bound to DNA or heparin.

### Evidence for CI-NTD:MOR interaction by gel filtration

**Figure 4:**
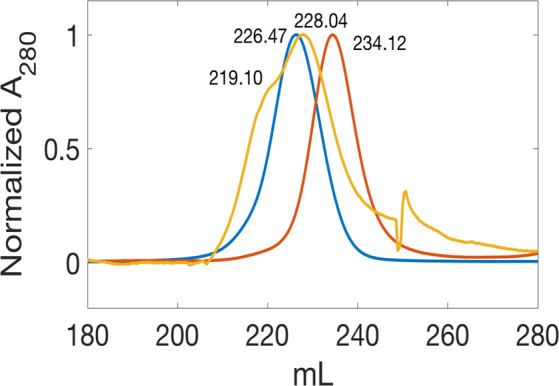
Comparison of free and bound CI-NTD89 and MOR. CI-NTD89 (blue), MOR (red), and a ~4:1 mixture of CI-NTD89:MOR (yellow) were purified using SEC SuperDex75 Prepgrad column. Normalized elution profiles were compared to verify complex formation. The two proteins, CI-NTD89 and MOR eluted as a single peak at 226.47 mL and 234.12 mL, respectively. In contrast the mixture (CI-NTD89:MOR) elute as two peaks at 219.10 mL and 228.04 mL, corresponding to M_w_ = 15.1 kDa and 12.8 kDa.

Guided by the pull-down experiments above, we attempted to purify the complex by gel filtration after mixing purified full length CI and MOR. However this could not be achieved reproducibly. Instead, we attempted a number of binding experiments trying to pull-down various truncated constructs of CI with GST-tagged MOR as bait. Some CI constructs seemed to be retained fairly consistently on affinity columns where GST-tagged MOR was bound, and co-eluted with MOR upon glutathione addition. Similarly, tagged MOR was sometimes retained and co-eluted with His-tagged CI constructs on IMAC columns. Unfortunately the results were not always consistent (data not shown) so we went on to investigate whether some of the available His-tagged CI constructs of different length could form complexes with MOR that were stable enough to be detected in gel filtration experiments. Accordingly, a construct of CI (CI-NTD_1–89_) including the N-terminal domain and the flexible linker, clearly formed a stable complex with MOR in gel filtration experiments (Fig. 4). After purification of GST-MOR by affinity chromatography, the GST linker had been cleaved off and MOR was further purified.

When the CI and MOR proteins were analyzed separately (Fig. 4), they migrated with molecular weights close to the expected 11 kDa for the CI-NTD89-His_6_ monomer (blue curve, 13 kDa estimated), and 8.4 kDa for the MOR monomer (red curve, 11 kDa estimated). It thus appears that they were both in the monomeric state at the concentrations used (38 μM and 10 μM, for CI-NTD89-His_6_ and MOR respectively). A mixture of CI-NTD89-His_6_ and MOR-His_6_ in 4:1 molar ratio (orange curve in Fig. 4), resulted in disappearance of the peak for MOR as expected and appearance of a peak with an estimated size around 15 kDa, which we interpret as a 1:1 complex of CI and MOR. The relative areas of the two peaks (the larger containing CI and the smaller containing CI and MOR) were estimated to be 1:3.9 in accordance with the expectation that a large fraction of MOR-His_6_ monomers had combined with CI-NTD89-His_6_ to form CI:MOR hetero-dimer complexes. There can be a number of explanations why our experiments with larger constructs of CI failed to show stable complexes with MOR under gel filtration conditions, and possibly our success with the CI-NTD89 protein could be due to the fact that its mobility rate is close to that of MOR and prevent separation of the two proteins during elution. At any rate, we interpret this experiment as definite *in vitro* proof of formation of a CI-NTD: MOR complex in the absence of DNA. Comparison of CI proteins from phages with and without MOR proteins previously identified a domain in the NTD region that was conserved only in CI repressors with MOR partners, and was suggested to contain the MOR interaction site^25^, but to our knowledge this is the first demonstration of a CI:MOR complex at the protein level.

### Evidence for CI:MOR and MOR:MOR interaction at high concentrations by chemical cross-linking *in vitro*

**Figure 5:**
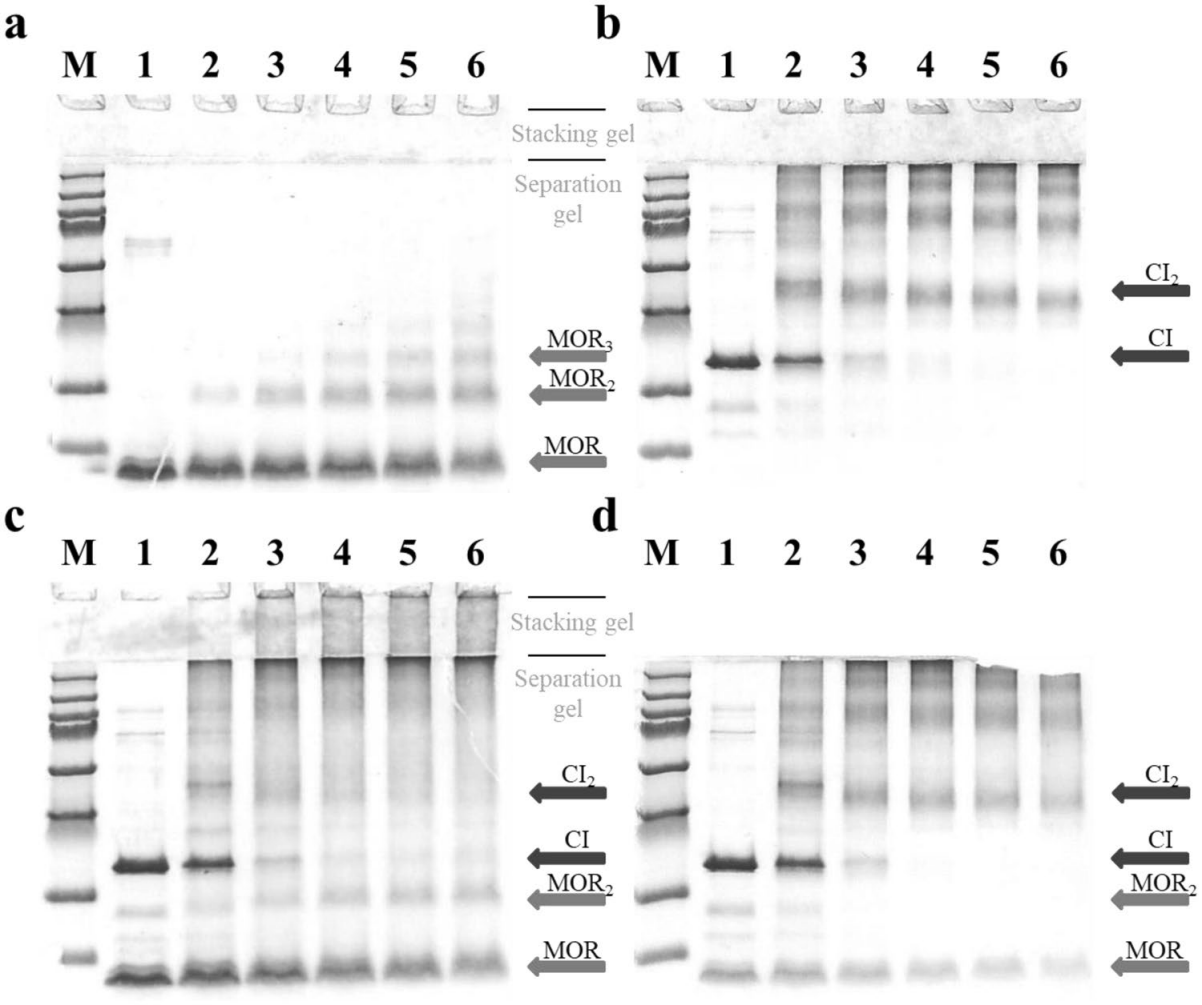
Identification of CI:CI, MOR:MOR, and CI:MOR interactions by chemical cross-linking *in vitro* Interacting proteins were chemical cross-linked with glutaraldehyde for 0, 2, 5, 10, 30, and 60 minutes (lanes 1 to 6). Purified protein in the cross-linking experiments was MOR alone (purified from AJ172) at 20 μM (**a**), CI alone (purified from AJ159) at 20 μM (**b**), MOR at 20 μM and CI at 20 μM (**c**), or MOR at 4 μM and CI at 20 μM (**d**). The cross linked samples were separated by SDS PAGE and coomassie stained.

To verify that also full length CI and MOR form a stable complex we attempted to cross-link the proteins chemically. To prevent cross-linking of the protein impurities, pooled fractions from gel filtration of the individual (untagged) proteins were used. Detection of specific cross-linked CI:MOR complexes did not prove to be easy, as we could not with certainty detect bands of the correct size after SDS PAGE analysis of the linked products (see Fig. 5c,d). Instead CI:MOR binding was shown indirectly by first detecting MOR-MOR adducts when MOR protein was cross-linked alone, and then showing that these adducts were converted to higher molecular weight adducts when CI was added together with MOR (see Fig. 5a,c,d). The formation of MOR:MOR (MOR_2_) complexes is a prerequisite for both model I and model II for the anti-immune CI:MOR complex, so the detection of a cross-linked MOR-MOR dimer complex is consistent with either model.

In the *in vitro* cross-linking assay where high concentrations of MOR at 20 μM and low salt concentration (100 mM) were used to compensate for a low binding affinity, cross-linked MOR:MOR adducts could be detected already after 2 minutes of incubation with glutaraldehyde (Fig. 5a, lane 2). The level of the cross-linked dimer species remained constant during the 60 min of incubation (lane 6). Larger cross-linked MOR adducts could be detected after 10 min of cross-linking (lane 4 to 6), but at a much lower level.

When purified CI protein at 20 mM was cross-linked in the absence of MOR, many different types of cross-linked CI-adducts could be detected (Fig. 5b). This was expected because in addition to the stable dimer conformation the CI protein is known to form larger oligomers such as hexamers in solution^3^. The dominant species appears to be a cross-linked CI-dimer in Fig. 5b, whose level decrease slightly between 2 min and 60 min cross-linking. In the presence of equimolar concentration of MOR (20 μM), the band corresponding to the CI_2_ adduct vanishes more rapidly with time (Fig. 5c), most likely due to the formation of larger cross-linked CI_2_-MOR adducts. No clear bands corresponding to such adducts are present in the separation gel, but a dense smear accumulate in the stacking gel above the separation gel at time points after 5 minutes of crosslinking, indicating the presence of large non-uniform crosslinked structures. Under these conditions, MOR protein appears to be in surplus resulting in the detection of MOR monomer and cross-linked MOR-MOR adducts. When the level of MOR is lowered to 4 μM (Fig. 5d), it becomes clear that bands corresponding to both MOR monomer, the MOR-MOR adduct, and the CI_2_ adduct all vanishes from 2 min to 60 min cross-linking. When the level the CI_2_ adduct is compared between Fig. 5c,d, it is clear that the MOR protein is responsible for the disappearance, most likely through a MOR:CI_2_ or MOR_2_:CI_2_ complex formation.

All and all the cross-linking experiments in Fig. 5 support both models I and II in directly detecting MOR:MOR interactions and indirectly showing CI:MOR interactions.

### A CI interaction domain on MOR

CI:MOR interactions require both a MOR interaction domain on CI and a CI interaction domain on MOR. We have demonstrated above previous suggestions that N-terminal domain of CI contains the MOR binding site. It has also been established by introduction of stop codons in the *mor* reading frame^3^ that a full size MOR protein is needed for bistability of switch plasmids, since 100% white transformants (in the immune state) were obtained when the nonsense mutants were introduced into TP901-1 switch plasmids. A stop codon at position 63 in the *mor* reading frame will result in a truncated MOR protein devoid of the last 10 amino acids including a conserved YF motif in the last (α5) helix which can be identified in the alignment of MOR proteins from the MOR-encoding phages harboring the O_M1_-O_M2_-O_R_-O_M3_ operator (Fig. 4b). The inability of this truncated MOR protein to engage in bistability suggests that the YF motif or possibly all of helix 5 is important for stability of the anti-immune operator complex.

### Two conceptual models for TP901-1 bi-stability in decision switching

**Figure 6:**
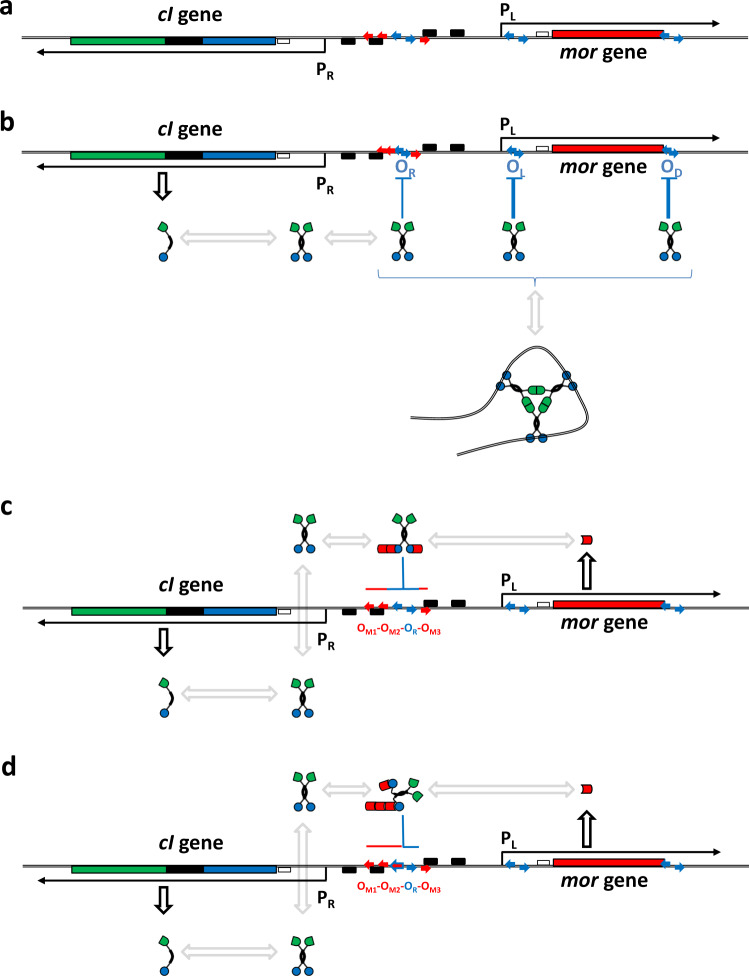
Two models for TP901-1 decision switching. (**a**) Physical map of the switch region. Genes are shown as colored boxes and labeled; promoter −10 and −35 elements are shown as black boxes; Binding sites are shown as colored arrows. Promoters and mRNA is shown as labeled black lines and arrows. (**b**) The immune state. CI repressor is shown with three domains: the NTD (DNA binding and MOR binding, blue); the CTD1 (dimerization hook domain black); and the CTD2 (multimerization domain, green). Between the NTD and the CTD1 a flexible linker ensures high freedom of the DNA binding angle in the CI_2_ dimer. (**c**) The anti-immune state according to Model I. MOR protein is shown as a single red domain, with the ability to bind DNA (O_M_ sites, red), to bind the CI-NTD domain and to form MOR:MOR dimers. (**d**) The anti-immune state according to Model II. MOR protein is shown as a single red domain, with the ability to bind DNA (O_RL_ and O_M_ sites, red) to bind the CI-NTD domain, and to form MOR:MOR dimers.

Above we have reported a number of experiments where the results appeared to support both models. To make it easier to see if any of the experiments could offer conclusions that point at one model over the other, we have outlined the possible stable endpoints of the decision process for the two contrasting models in Fig. 6(c,d). Three protein domains are highlighted for the CI protein, the DNA and MOR binding N-terminal domain in blue, the hook forming dimerization domain in black, and the multimerization domain in green. It is not clear whether the multimerization domain function as a secondary low affinity dimerization domain promoting ring-formation into hexamers or higher multimers (as suggested in Fig. 6b), or whether the C-terminal forms hexamers with a more complex interaction domain. Compared to a previous model^25^ we have introduced the O_M_ sites and specified how an anti-immune repressing complex may be bound to the composite O_M1_-O_M2_-O_RL_- O_RR_-O_M3_ site in the anti-immune state.

As explained previously, Model I presupposes that MOR binds to a part of the N-terminal domain which faces outward if the CI_2_ dimer is bound to the O_R_ operator. Model II presupposes that the binding is on the opposite side of the N-terminal domain.

Figure 6a shows the geometry of the minimal switch region, which is able to perform decision switching, and 6b shows the outcome of the immune switching phenotype, common to all three models. CI monomers expressed from the *cI* gene dimerize and bind to O_R_, O_L_, and O_D_, either before or after hexamer formation.

Logically, model I (Fig. 6c) is the most straight-forward consequence of the switch geometry, because it would enable CI_2_ to bind to O_R_ both under immune and anti-immune conditions. As a MOR:CI_2_:MOR complex the central CI_2_ would bind to the central O_R_ site in the composite O_M2_-O_R_-O_M3_ operator site with a lowered affinity which is then compensated by the binding of the flanking MOR molecules to O_M2_ and O_M3_. Furthermore, the MOR:CI_2_:MOR complex would also have reduced binding affinity for O_L_ and O_D_, accounting for the de-repression of the P_L_ promoter under anti-immune conditions. Model I was our model of choice until we started to interpret all data according to the model and tried to find alternative models that could fit the data.

## Conclusion

We have presented two models for establishment of the anti-immune repression of the P_R_ promoter in TP901-1 and related bacteriophages against bacteria of *Lactococcus, Streptococcus, Enterococcus*, and *Staphylococcus* species. The two models differ in the mechanism behind the anti-immune repression of the P_R_ promoter, which leads to the lytic cycle of the intact bacteriophage. A number of experiments point at the importance of the postulated O_M_ sites that form the basis for both models, and no experiments are in direct contradiction with any of the models or directly point at one over the others. We will therefore conclude that none of our working models explaining TP901-1 decision switching have been falsified, and further detailed structural and functional characterization of the CI:MOR complex will be needed to conclude which of the models offer the best description.

## Methods

### Bacterial strains, transformation and growth conditions

The bacterial strains used in this work are listed in Table S1 in supplementary materials. MG1363 was propagated at 30 °C in GM17 media (M17 broth from Oxid Limited supplemented with 0.5% glucose)^29^. Erythromycin (erm) or chlorampenicol (cam) at 5 μg/ml, was added to the media when appropriate. *L. lactis* strains were transformed by electroporation as previously described^3^. Screening on 5-bromo-4-choloro-3-indolyl-β-D-galactopyranoside (X-gal) plates were performed at a concentration of 90 to 150 μg/ml. *Escherichia coli* strains was grown at 37 °C with agitation in lysogenization broth (LB) media^30^. Bacto Agar was used at 1.5% (wt/vol) in solid media. 100 μg/ml ampicillin and 8 μg/ml tetracycline was added to the media when appropriate. Electrocompetent *E. coli* was obtained by growth in LB media to OD450 0.8 followed by several washes in 10% glycerol. The cells were stored at –80 °C until electroporation (200 Ohm, 25 μFD, 2.0 kV).

### DNA techniques

Primers were supplied by TAG Copenhagen A/S Denmark or DNA Technology A/S Denmark. The GFX^TM^ PCR DNA and Gel Band Purification Kit were supplied by GE Healthcare Denmark. T4 DNA ligase, restriction enzymes and buffer systems were supplied by Fermentas GmbH Germanys and used as recommend by the supplier. Plasmid DNA from *E. coli* was isolated using the Qiagen Plasmid kit as described the manufacturer (Qiagen). DNA sequencing was performed by Eurofins MWG Operon, Germany, or Macrogen, Korea.

### Construction of plasmids

The plasmids used in this study are listed in Table S2 in supplementery materials. Unless otherwise noted, Inserts were amplified by PCR from purified TP901-1 or pMAP50 DNA using the primers and vectors indicated in the description. Nucleotide sequence of primers are found in Table S3. All constructs were verified by sequencing (Macrogen).

### Localized random mutagenesis of O_M_ sites

The relevant fragments were amplified by PCR using the degenerate primers indicated in Table S3) and digested with HindIII and PstI. Digested PCR fragments were inserted into HindIII and PstI digested pLB85. The correct nucleotide sequence of the resulting fusion plasmids pJT2 to pJT13 and pMK1216 to pMK1217 were verified before insertion of the plasmids into the chromosomes of JT2 and LB504. Insertion into strain JT2 required co-transformation with plasmid pLB65 encoding the TP901-1 integrase.

### Site directed mutagenesis of the *mor* gene

Site-specific mutants were introduced in the *mor* gene using the QuickChange Site-Directed Mutagenesis Kit from Stratagene. The reactions were carried out as described by the manufacturer. Plasmid pAB223 was used as DNA template and plasmid pMAP109 containing alanine codons in postion 36 and 39 in *mor* was constructed using primers MOR36A39A.for and MOR36A39A.rev. The construct was verified by sequencing.

### Protein purification and pull down experiment using unspecific DNA binding

Partial purification of wild type CI protein took advantage of the salt dependent unspecific DNA binding properties of the repressor. For over-expression of CI we used *E. coli* strain AJ159, containing *cI* in the expression vector pUHE23-2. AJ159 was grown in LB broth (750 ml) containing 100 μg/ml ampicillin and 8 μg/ml tetracycline at 37 °C with agitation to an optical density of 0.6–0.7, induced with isopropyl-1-thio-β-D-galactopyranoside to a final concentration of 1 mM, and grown for further 2–3 hours. Cells were harvested by centrifugation, washed with buffer A (see below), and the cell pellet was stored at −20 °C until used.

*E. coli* strains expressing wild type MOR (AJ172) or MOR[K36A D39A] mutant (MP1047) from the *mor* genes inserted in the expression vector pUHE23–2 were grown in LB broth (750 ml) containing 100 μg/ml ampicillin and 8 μg/ml tetracycline at 37 °C with agitation to an optical density of 0.6–0.7. After this, the culture was induced with isopropyl-1-thio-β-D-galactopyranoside to a final concentration of 1 mM, and grown for further 2–3 hours. Cells were harvested by centrifugation, washed with buffer A (see below), and the cell pellet was stored at −20 °C until used.

The cell pellet was resuspended in 10 ml of buffer A (20 mM Tris/HCl pH 8, 1 mM EDTA, 5 mM MgCl2, 0.1 M NaCl) and sonicated on ice. Cell debris and CI protein was sedimented by centrifugation in a Sorvall SS34 rotor at 13.000 rpm for 30 minutes. The pellet was resuspended in buffer B, a high salt buffer, (20 mM Tris/HCl pH 8, 1 mM EDTA, 5 mM MgCl2, 1 M NaCl) and centrifuged using a Sorvall SS34 rotor at 13.000 rpm for 30 minutes.

Pull down experiments used extracts of MOR from AJ172 or of MOR[K36A D39A] from MP1047, which were either subjected directly to trials of purification by unspecific DNA binding, or by mixing of the extracts with extracts from AJ172 cells prior to purification by unspecific DNA binding.

### Expression, purification and detection of complex-formation by gel filtration of tagged CI and MOR derivatives for detection of CI:MOR interaction by gel filtration

*E.coli* strain BL21(dea) was transformed with either pGEX4-2 containing GST-MOR (1–72) or pET30a(+) containing CI-NTD (1–80) + linker (81–89). Pre cultures in 10 mL LB medium were used to inoculate 1 L M9 medium containing Glucose and NH_4_Cl for GST-MOR and 1 L LB medium expression cultures for CI-NTD89. At OD_600_ = 0.6 expression were started by adding 0.2 mM or 1 mM IPTG as final concentration for GST-MOR and CI-NTD89, respectively. After 20 hours cells were harvested, sonicated and supernatant was collected. The protein of interest was immobilised by loading supernatant onto a GST-Trap equilibrated with PBS pH 7.4 (GE HealthCare) (GST-MOR) or His-Trap eqillibrated with 20 mM Tris, 100 mM NaCl pH 7.4 buffer (GE HealthCare) (CI-NTD89). Liberation of MOR from the GST linker took advantage of a thrombin cleavage site between GST and MOR. After washing the GST-Trap column with PBS, cleavage was performed by injecting 1 mL PBS with 50 unit of Thrombin. After 16 hours cleavage at room temperature, cleaved MOR were eluted with PBS and purified further by size exclusion chromatography (SEC) with 20 mM Tris, 100 mM NaCl pH 7.4 as eluent. CI proteins were eluted with 20 mM Tris, 100 mM NaCl, 250 mM Imidazole, pH 7.4, and further purified by SEC with same eluent as MOR.

A Superdex 75 Prep Grad column was equilibrated with 20 mM Tris, 100 mM NaCl, pH 7.4, and gel filtration was used to quantify the native size of purified proteins: MOR, NTD89 and MOR:NTD89 mixed in 1:4 ratio.

For calibration of the column 0.25 mg Thyroglobulin (725 kDa), Ferritin (391 kDa), Catalase (202 kDa), Aldolase (171 kDa), Albumin (63.5 kDa) or Ribonuclease A (15.6 kDa) (Amersham Biosciences) was used. The calibration proteins were run in duplex. Protein elution from the column was monitored at 280 nm.

### Chemical cross-linking of complexes containing purified CI and MOR

Chemical cross-linking experiments with CI and MOR was performed with pooled fractions from Superdex gel filtration containing 450 μM and 75 μM CI and MOR, respectively.

CI and/or MOR protein was added at final concentrations of CI at approximately 20 μM, and MOR at 4 μM or 20 μM in high stringency binding buffer (20 mM HEPES pH 7.5 200 mM NaCl). At time zero glutaraldehyde was added to the protein sample to a final concentration of 0.05% followed by incubation at room temperature. Samples were removed after 2, 5, 10, 30 or 60 minutes and prepared for analysis by SDS-PAGE. The molecular weight of the protein bands was calculated using a low range molecular weight marker (LMW) and a high range molecular weight marker (HMW) from Biorad.

## Supplementary information

Supplementary file.

## References

[1] Oppenheim AB, Kobiler O, Stavans J, Court DL, Adhya S (2005). Switches in Bacteriophage Lambda Development. Annu. Rev. Genet..

[2] Madsen PL, Johansen AH, Hammer K, Brøndsted L (1999). The genetic switch regulating activity of early promoters of the temperate lactococcal bacteriophage TP901-1. J. Bacteriol..

[3] Pedersen M, Hammer K (2008). The Role of MOR and the CI Operator Sites on the Genetic Switch of the Temperate Bacteriophage TP901-1. J. Mol. Biol..

[4] Alsing A, Pedersen M, Sneppen K, Hammer K (2011). Key players in the genetic switch of bacteriophage TP901-1. Biophys. J..

[5] Rasmussen, K. K. *et al*. Structural and dynamics studies of a truncated variant of CI repressor from bacteriophage TP901-1. *Sci. Rep*. **6**, (2016). 10.1038/srep29574 PMC4941734 27403839

[6] Breüner A, Brøndsted L, Hammer K (1999). Novel organization of genes involved in prophage excision identified in the temperate lactococcal bacteriophage TP901-1. J. Bacteriol..

[7] Kenny JG (2006). Characterization of the lytic-lysogenic switch of the lactococcal bacteriophage Tuc2009. Virology.

[8] Frandsen KH (2013). Binding of the N-terminal domain of the lactococcal bacteriophage tp901-1 ci repressor to its target DNA: A crystallography, small angle scattering, and nuclear magnetic resonance study. Biochemistry.

[9] Rasmussen Kim K., Varming Anders K., Schmidt Simon N., Frandsen Kristian E. H., Thulstrup Peter W., Jensen Malene Ringkjøbing, Lo Leggio Leila (2018). Structural basis of the bacteriophage TP 901‐1 CI repressor dimerization and interaction with DNA. FEBS Letters.

[10] Pedersen M, Kilstrup M, Hammer K (2006). Identification of DNA-binding sites for the activator involved in late transcription of the temperate lactococcal phage TP901-1. Virology.

[11] Nakanishi H, Pedersen M, Alsing AK, Sneppen K (2009). Modeling of the Genetic Switch of Bacteriophage TP901-1: A Heteromer of CI and MOR Ensures Robust Bistability. J. Mol. Biol..

[12] Graña D, Gardella T, Susskind MM (1988). The effects of mutations in the ant promoter of phage P22 depend on context. Genetics.

[13] Shearwin KE, Brumby AM, Egan JB (1998). The tum protein of coliphage 186 is an antirepressor. J. Biol. Chem..

[14] Brumby AM, Lamont I, Dodd IB, Egan JB (1996). Defining the SOS operon of coliphage 186. Virology.

[15] Velleman M, Heinzel T, Schuster H (1992). The Bof protein of bacteriophage P1 exerts its modulating function by formation of a ternary complex with operator DNA and C1 repressor. J. Biol. Chem..

[16] Schaefer TS, Hays JB (1990). The bof gene of bacteriophage P1: DNA sequence and evidence for roles in regulation of phage c1 and ref genes. J. Bacteriol..

[17] Heinrich J, Velleman M, Schuster H (1995). The tripartite immunity system of phages P1 and P7. FEMS Microbiol. Rev..

[18] Yu A, Haggard-Ljungquist E (1993). The Cox protein is a modulator of directionality in bacteriophage P2 site- specific recombination. J. Bacteriol..

[19] Eriksson JM, Haggård-Ljungquist E (2000). The multifunctional bacteriophage P2 Cox protein requires oligomerization for biological activity. J. Bacteriol..

[20] Dodd IB, Reed MR, Egan JB (1993). The Cro‐like Apl repressor of coliphage 186 is required for prophage excision and binds near the phage attachment site. Mol. Microbiol..

[21] Bolotin A (2001). The complete genome sequence of the lactic acid bacterium lactococcus lactis ssp. lactis IL1403. Genome Res..

[22] Gasson MJ (1983). Plasmid complements of Streptococcus lactis NCDO 712 and other lactic streptococci after protoplast-induced curing. J. Bacteriol..

[23] Altschul SF (1997). Gapped BLAST and PSI-BLAST: A new generation of protein database search programs. Nucleic Acids Res..

[24] Little JW (1984). Autodigestion of lexA and phage lambda repressors. Proc. Natl. Acad. Sci. USA.

[25] Pedersen M, Ligowska M, Hammer K (2010). Characterization of the CI repressor protein encoded by the temperate lactococcal phage TP901-1. J. Bacteriol..

[26] Hayes F, Daly C, Fitzgerald GF (1990). Identification of the Minimal Replicon of Lactococcus lactis subsp. lactis UC317 Plasmid pCI305. Appl. Environ. Microbiol..

[27] Brøndsted L, Hammer K (1999). Use of the integration elements encoded by the temperate lactococcal bacteriophage TP901-1 to obtain chromosomal single-copy transcriptional fusions in Lactococcus lactis. Appl. Environ. Microbiol..

[28] Johansen AH, Brøndsted L, Hammer K (2003). Identification of operator sites of the CI repressor of phage TP901-1: Evolutionary link to other phages. Virology.

[29] Terzaghi BE, Sandine WE (1975). Improved medium for lactic streptococci and their bacteriophages. Appl. Microbiol..

[30] Bertani G (1951). Studies on lysogenesis. I. The mode of phage liberation by lysogenic Escherichia coli. J. Bacteriol..

