# Supplementary material for: Repression of the lysogenic PR promoter in bacteriophage TP901-1 through binding of a CI-MOR complex to a composite OM-OR operator
Source: Sci Rep. 2020 May 26;10:8659. doi: 10.1038/s41598-020-65493-0 (PMC7250872; doi:10.1038/s41598-020-65493-0)
Supplement: Supplementary file 1 — Supplementary file. [file 41598_2020_65493_MOESM1_ESM.docx]

**Repression of the lysogenic P_R_ promoter in bacteriophage TP901-1 through binding of a CI-MOR complex to a composite O_M_-O_R_ operator.**

**Supplementary materials**

Margit Pedersen^1^, Jesper Tvenge Neergaard^2^, Johan Cassias^2^, Kim Krighaar Rasmussen^3^, Leila Lo Leggio^3^, Kim Sneppen^4^, Karin Hammer^2^ & Mogens Kilstrup^2^*

^1^University of Copenhagen, Department of Biology, Copenhagen, DK2200, Denmark

^2^Technical University of Denmark, Department of Biotechnology and Biomedicine, Lyngby, DK2800, Denmark

^3^University of Copenhagen, Department of Chemistry, Copenhagen, DK2200, Denmark

^4^University of Copenhagen, Center for Models of Life, Copenhagen, DK2200, Denmark

*Mogens Kilstrup, email mki@bio.dtu.dk

**Table S1. Strain list**

| Strain designation | Species | Relevant genotype and phenotype | Reference or origin |
| --- | --- | --- | --- |
| MG1363 | *L. lactis* | Plasmid and prophage cured laboratory strain | ^1^ |
| LB504 | *L. lactis* | MG1363/pLB95 (encodes TP901-1 integrase, needed for integration of pLB86 derivatives) | ^2^ |
| JT2 | *L. lactis* | MG1363/pAB223 (TP901-1 switch expressing anti-immune CI:Mor complex) | This work |
| XL1 Blue | *E.coli* | *recA1 endA1 gyrA96 thi-1 hsdR17 supE44 relA1 lac* [F´ *proAB lacIq Z∆M15* Tn*10* (Tet^r^)]. | Stratagene |
| MC1061 | *E. coli* | K12, Lambda^-^ *araD139* *Δ(araA-leu)7697* *Δ(lac)X74* *galK16* *galE15*(GalS) *mcrA0* *relA1* *rpsL150*(strR) *spoT1* *mcrB1* *hsdR2* | ^3^ |
| AJ159 | *E. coli* | XL1 Blue/pAJ134 (expression of wild type CI repressor, from the pUHE23-2 platform) | Anette Johansen, PhD thesis DTU, 2000 |
| AJ172 | *E. coli* | XL1 Blue/pAJ144 (expression of wild type MOR repressor, from the pUHE23-2 platform) | Anette Johansen, PhD thesis DTU, 2000 |
| AJ189 | *L. lactis* | MG1363/pAJ149 (*P_L_-lacLM* fusion in pAK80, containing the full intergenic region between *cI* and *mor*, coordinates 3142 to 3458, with expression of no functional CI or MOR proteins). Amp^R^ | ^4^ |
| MP1034 | *E. coli* | XL1 Blue/pMAP148 | This work |
| MP1047 | *E. coli* | XL1 Blue/pMAP158 | This work |

**Table S2. Plasmid list**

| plasmid designation | Description | Relevant genotype and phenotype | Reference or origin |
| --- | --- | --- | --- |
| pAK80 | Reporter fusion plasmid replicating in *L. lactis* | Promoterless *lacLM* reporter gene | ^5^ |
| pLB86 | Reporter fusion plasmid integrating in *L. lactis* at *attB^TP901-1^* | Promoterless *lacLM* reporter gene | ^2^ |
| pCI3340 | *E. coli-L. lactis* shuttle vector | Canamycin resistance | ^6^ |
| pUHE23-2 | Expression plasmid |  | H. Bujard |
| pGEX4-2 | Expression plasmid |  | Addgene |
| pET30a(+) | Expression plasmid |  | Addgene |
| pML32 | Plasmid selected after insertion mutagenesis, showing normal P_L_ but decreased P_R_ repression | Insertion mutant (CI[D88<insertion CLNTV>S89]) of plasmid pAJ80, containing the region {*cI*🡨P_R_ O_M1_ O_M2_ O_R_ O_M3_ P_L_🡪} coordinates 2622 to 3332, inserted in pCI372 | ^7^ |
| pAB223 | Switch plasmid: TP901-1 switch region inserted in pCI3340 | Full switch region from TP901-1, capable of decision switching. No reporter genes present  (Cam^R^) | ^8^ |
| pMAP50 | Switch plasmid: TP901-1 P_L_ *lacLM* fusion in pAK80 | {*cI*🡨P_R_ O_M1_ O_M2_ O_R_ O_M3_ P_L_🡪O_L_ *mor* O_D_} *lacLM* | ^9^ |
| pMAP91 | Switch plasmid: TP901-1 P_L_ *lacLM* fusion in pAK80 (mutated O_RR_ site) | {*cI*🡨P_R_ O_M1_ O_M2_ [O_RR_: AATTCAT to GGGATCC] O_M3_ P_L_🡪O_L_ *mor* O_D_} *lacLM* | ^10^ |
| pMAP109 | Switch plasmid: TP901-1 switch region inserted in pCI3340 (mutated MOR HTH) | Full switch region from TP901-1. No reporter genes present. (Cam^R^). Derivative of pAB223 with MOR[K36A D39A] | This work |
| pMAP158 | TP901-1 *mor* expression fusion pUHE23-2 (Mutated MOR HTH) | IPTG induced production of MOR[K36A D39A] | This work |
| pMAP148 | TP901-1 *cI* expression fusion in pQE70 (PCR with primers CISphI + CIBglII and template pML32) | IPTG induced production of CI[D88<insertion CLNTV>S89]-ArgCysHis_6_ | This work |
| pJT2 | TP901-1 P_R_ promoter fusion (PCR with primers MK661+MK663) in pLB86 | *lacLM*{🡨P_R_ O_M1_ O_M2_ O_R_ O_M3_ P_L_🡪O_L_} | This work |
| pJT3 | TP901-1 P_R_ promoter fusion (PCR with primers MK661 + MK663 and pMAP91 as template) in pLB86 | *lacLM*{🡨P_R_ O_M1_ O_M2_ [O_RR_: AATTCAT to GGGATCC] O_M3_ P_L_🡪O_L_} | This work |
| pJT5 | TP901-1 P_R_ promoter fusion (PCR with primers MK661+MK665) in pLB86 | *lacLM*{🡨P_R_ O_M1_ O_M2_ O_R_ O_M3_} | This work |
| pJT6 | TP901-1 P_R_ promoter fusion (PCR with primers MK661+MK666) in pLB86 | *lacLM*{🡨P_R_ O_M1_ O_M2_ O_R_} | This work |
| pJT13 | TP901-1 P_R_ promoter fusion (PCR with primers MK661+MK684) in pLB86 | *lacLM*{🡨P_R_ [O_M1_ AAA to TCC] O_M2_ O_R_ O_M3_} | This work |
| pMK1216 | TP901-1 P_R_ promoter fusion (PCR with primers MK661+MK683) in pLB86 | *lacLM*{🡨P_R_ O_M1_ [O_M2_ AAAA to CGTG] O_R_ O_M3_} | This work |
| pMK1217 | TP901-1 P_R_ promoter fusion (PCR with primers MK661+MK682) in pLB86 | *lacLM*{🡨P_R_ O_M1_ O_M2_ O_R_ [O_M3_ AA to GT]} | This work |

**Table S3. Primer list**

| Primer designation | Nucleotide sequence |
| --- | --- |
| MK661 | AAAACTGCAGCATAAAAACCTCTTTTTTATTTTG |
| MK663 | AAAAAAGCTTGTTTCTCCTTTCTTTCAGTTCAC |
| MK665 | AAAAAAGCTTTTCTGTCAACAAAAAAGTTCACGAA |
| MK666 | AAAAAAGCTTAGTTCACGAAATATGAATTTTTTTGTTG |
| MK682 | AAAAAAGCTTTGTCAACA**NNNN**AGTTCACGAAATATGAATTTTTTTG |
| MK683 | AAAAAAGCTTTGTCAACAAAAAAGTTCACGAAATATGAATTT**NNNN**GTTGAACTTTTGTTCAAGATGC |
| MK684 | AAAAAAGCTTTGTCAACAAAAAAGTTCACGAAATATGAATTTTTTTGTTGAAC**NNNN**GTTCAAGATGCGCTATAATC |
| CISphI | GGGGGCATGCAAACTGATACTAGTAATAGG |
| CIBglII | GGGGGGAGATCTTAAATCGCCTTTATCAATTTT |

**Growth phase dependence of P_R_ promoter activity necessitates analysis under balanced conditions in a limited range of optical densities.**

Before the peculiarities of the TP901-1 P_R_ promoter were revealed, the reproducibility of P_R_ fusion data was problematic, but it was soon realized that the specific β-galactosidase activity, defined as the total activity per ml per OD_450_, increased at high cell densities during the exponential growth phase. Since the dependence could influence the repression measurements, we monitored the β-galactosidase expression from a native P_R_-*lacLM* fusion during balanced growth. The cultures were inoculated at a low density from a balanced pre-culture that had grown exponentially for at least ten generations in GSAL medium. After quantifying the β-galactosidase activity at various time points, two different plots of the activities were made (figure S1). The first curve was plotted with the specific activity as a function of the cell density using filled symbols. In this plot, a high initial specific activity at the lowest cell densities (OD_450_=0.05) was seen to be diluted out in the first three doublings, reaching a low specific activity at OD_450_=0.4. Above this cell density the specific activity increased until OD_450_=1.2. In the second curve (the differential plot, shown with open symbols) the total β-galactosidase activity per volume was plotted as a function of the optical density. Here the slope of the curve gives the differential synthesis rate, defined as Δ(activity per ml)/Δ(cell per ml), which under balanced conditions is equal to the specific activity. From figure S1 it appears like the differential synthesis rate (the P_R_ promoter activity) is constant until OD_450_=0.5, where the curve deviates from the stipulated line. Above 0.5 the slope increases slightly as a function of the cell density without reaching a constant differential synthesis rate.


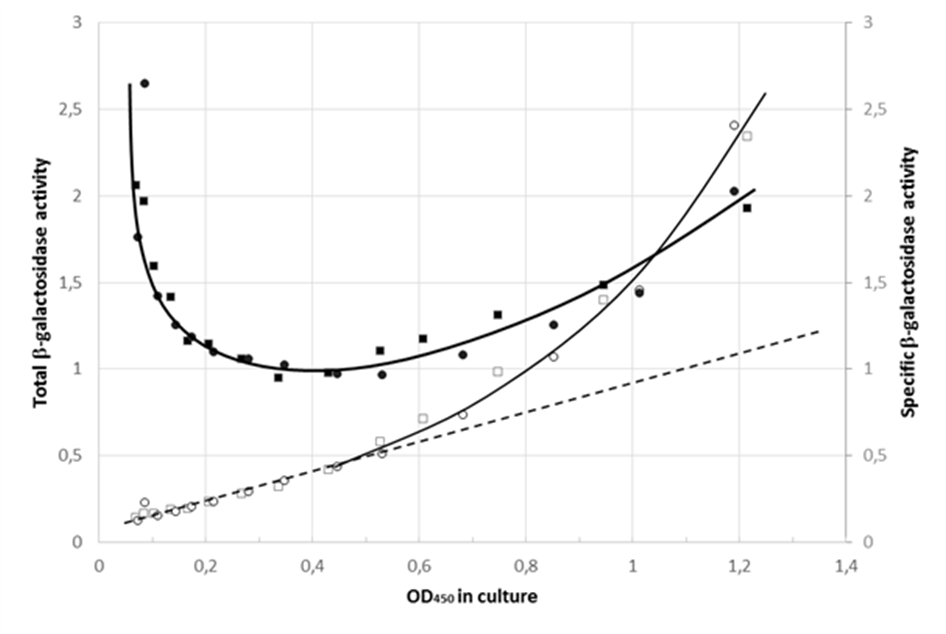


**Figure S1. Dependence of P_R_ promoter activity on optical density.** β-galactosidase activity was measured at various time points under balanced conditions, after inoculation from a balanced pre-culture to OD600 = 0.05. The activity was plotted as a function of the optical density of the culture, measured at a wavelength of 450nm. Filled symbols, Specific activity (units per OD450, right axis); Open symbols, total activity (units per ml, left axis).

From figure S1 it is clear that the P_R_ promoter activity is not completely linear from 0.25 to 0.75 and our determinations may have resulted in a slight overestimation of the P_R_ promoter activity compared to the linear relation at low cell density, but this was judged acceptable. To be certain that identical conclusions could be drawn whether one or the other method was used, we have also included the specific β-galactosidase activities (total enzyme activity/OD_450_) for all samples collected at OD_450_ values between 0.25 and 0.6 (Table S4). Mean values and standard deviations of these activities were somewhat different from the values obtained from the differential synthesis rate, but all conclusions about changes in P_R_ repression for the different mutations were substantiated.

**Table S4.** **Values from alternative mathematical extraction methods.** Expression levels from P_R_-*lacLM* reporter fusions, shown in Table 4 and 5. See text for details.

|  | Method of mathematical extraction | β-galactosidase activity | | |
| --- | --- | --- | --- | --- |
| Fusion plasmid |  | P_R_ level in JT2 (SD) | P_R_ level in LB504 (SD) | Repression fold |
| pJT2 | All data used  Data from low cell density  Differential plot | 0.30 (0.082)  0.32 (0.082)  0.17 (0.086) | 5.8 (1.9)  5.4 (2.0)  6.6 (4.2) | 19  17  **39** |
| pJT3 | All data used  Data from low cell density  Differential plot | 0.73 (0.47)  0.79 (0.48)  0.45 (0.53) | 2.4 (0.94)  2.5 (1.1)  1.8 (1.7) | 3.3  3.2  **4.0** |
| pJT5 | All data used  Data from low cell density  Differential plot | 0.39 (0.095)  0.41 (0.11)  0.20 (0.069) | 4.8 (0.98)  5.0 (0.53)  4.3 (3.4) | 12  12  **22** |
| pJT13 | All data used  Data from low cell density  Differential plot | 2.0 (0.77)  1.8 (0.87)  3.6 (0.31) | 3.8 (1.7)  3.4 (2.1)  5.9 (0.097) | 1.9  1.9  **1.6** |
| pMK1216 | All data used  Data from low cell density  Differential plot | 0.56 (0.11)  0.59 (0.12)  0.37 (0.072) | 3.2 (0.41)  3.2 (0.47)  2.8 (0.52) | 5.7  5.4  **7.6** |
| pMK1217 | All data used  Data from low cell density  Differential plot | 0.99 (0.28)  1.0 (0.35)  0.82 (0.23) | 2.8 (0.65)  2.9 (0.79)  2.1 (0.48) | 2.8  2.9  **2.6** |
| pJT6 | All data used  Data from low cell density  Differential plot | - 1. (0.34)   2. (0.35)   0.46 (0.24) | 1.9 (0.59)  2.1 (0.64)  1.3 (0.91) | 1.7  1.8  **2.8** |

References

1. Gasson, M. J. Plasmid complements of Streptococcus lactis NCDO 712 and other lactic streptococci after protoplast-induced curing. *J. Bacteriol.* **154**, 1–9 (1983).

2. Brøndsted, L. & Hammer, K. Use of the integration elements encoded by the temperate lactococcal bacteriophage TP901-1 to obtain chromosomal single-copy transcriptional fusions in Lactococcus lactis. *Appl. Environ. Microbiol.* **65**, 752–758 (1999).

3. Casadaban, M. J. & Cohen, S. N. Analysis of gene control signals by DNA fusion and cloning in Escherichia coli. *J. Mol. Biol.* **138**, 179–207 (1980).

4. Johansen, A. H., Brøndsted, L. & Hammer, K. Identification of operator sites of the CI repressor of phage TP901-1: Evolutionary link to other phages. *Virology* **311**, 144–156 (2003).

5. Israelsen, H., Madsen, S. M., Vrang, A., Hansen, E. B. & Johansen, E. Cloning and partial characterization of regulated promoters from Lactococcus lactis Tn917-lacZ integrants with the new promoter probe vector, pAK80. *Appl. Environ. Microbiol.* **61**, 2540–2547 (1995).

6. Hayes, F., Daly, C. & Fitzgerald, G. F. Identification of the Minimal Replicon of Lactococcus lactis subsp. lactis UC317 Plasmid pCI305. *Appl. Environ. Microbiol.* **56**, 202–209 (1990).

7. Pedersen, M., Ligowska, M. & Hammer, K. Characterization of the CI repressor protein encoded by the temperate lactococcal phage TP901-1. *J. Bacteriol.* **192**, 2102–2110 (2010).

8. Madsen, P. L., Johansen, A. H., Hammer, K. & Brøndsted, L. The genetic switch regulating activity of early promoters of the temperate lactococcal bacteriophage TP901-1. *J. Bacteriol.* **181**, 7430–7438 (1999).

9. Pedersen, M., Lo Leggio, L., Grossmann, J. G., Larsen, S. & Hammer, K. Identification of Quaternary Structure and Functional Domains of the CI Repressor from Bacteriophage TP901-1. *J. Mol. Biol.* **376**, 983–996 (2008).

10. Pedersen, M. & Hammer, K. The Role of MOR and the CI Operator Sites on the Genetic Switch of the Temperate Bacteriophage TP901-1. *J. Mol. Biol.* **384**, 577–589 (2008).
